# Fractures in Warfare

**Published:** 1919-01

**Authors:** 


**Fractures in Warfare.** By Sir ARBUTHNOT LANE. *The Practitioner*, October, 1918.

The fractures which occur in warfare may be conveniently classified under these headings :

1. Simple fractures.
2. Compound fractures not produced by projectiles.
3. Compound fractures produced by projectiles.

As regards simple fractures, this war's experience might lead the observer to conclude that few injuries have been treated so unsatisfactorily as these. This appears to be due either to a want of familiarity with the mechanics of fractures, or to such an imperfect operative technic as to suggest but little knowledge of the important details which a surgeon should possess, when he is called upon to operate on fractured bones.

The rule that should guide a surgeon in having recourse to operation in any particular case of fracture seems to be perfectly simple. If he is satisfied that he cannot, by manipulation under an anesthetic, prolonged extension, etc., replace the broken fragments of a bone in a simple fracture in accurate apposition, and if he believes that failure to restore continuity of the fragments is likely to interfere with the wage-earning capacity of the individual or with the requisite usefulness of the limb to the patient, it is his duty to operate and to restore the bone to its original form and function.

Operations for disability resulting from non-union of fragments in a simple fracture, or from overlapping of fragments, or from union of the fragments so that their long axes do not correspond, are at times very difficult. This is particularly the case when the fracture is in the vicinity of important nerves.

In the case of fractured bones of old standing, in which an operation is performed for non-union or axal deformity, it is well to remember that the period required to ensure firm bony union between the coapted fragments is very much longer than in operations performed for recent fractures.

As regards compound fractures not produced by projectiles, the surgeon must obtain accurate apposition of the fragments, if he possibly can, by manipulation, traction and splinting. These fractures vary in character in their degree of infection, sometimes being hardly, if at all, more serious than simple fractures, while at other times they have associated with them all the risks of a compound fracture produced by a projectile.

In the case of compound fractures produced by projectiles, while it is most important to excise any damaged soft parts, and to remove obviously useless fragments of bone at as early a date as possible, no special treatment of the bone is called for, other than extension sufficient to bring the fragments into apposition, etc.

Owing to the presence of a wound, often of considerable size, in these compound fractures, there is a free exit for the effused blood and the inflammatory exudation which is poured out at a very early period. Consequently the soft parts, which form the ties in the length of the limb, are not shortened nearly as much as in simple fractures, and afford comparatively little resistance to the extension of the fragments on one another, so that overlapping of the broken ends is readily avoided if suitable means are adopted.

Only in very exceptional circumstances is it advisable to fix fragments of broken bones together by means of plates and screws while the wound is foul.

It is advisable to postpone operative interference until the wounds have healed for a considerable time and the tissues are, in all probability, free of organisms. This can usually be determined with reasonable certainty.

Should there be any definite suspicion of presence of latent sepsis, irrigation by Carrel's or similar method must be adopted at once. If not, the wound should be closed completely at the time of operation.

Every attempt should be made to avoid any shortening of the limb, or to reduce it to a minimum.

It is most important that the muscles and joints, which are the fractured bone, shall be moved voluntarily by the patient as soon as possible after the operation, in order to avoid that stiffness and limitation of movement that often complicate these fractures. This is especially the case in the joints of the knee, ankle, and foot.

It is obvious that such early treatment cannot be adopted when the fragments are very fragile and the screws insecure.

Providing no strain shall be exerted on the junction likely to develop non-union, the sooner the patient who has been operated on for fracture of one or more long bones of the leg is got up and about, the more bone will be deposited and the more rapid will be the repair at the seat of fracture.

Should the interval between the fragments be so considerable that union is not likely to take place, the fragments should be secured in perfect alignment by a plate fixed vertically behind the centre of the shaft.

The essence of success depends on the absolute immobilization of the fragments of the shaft on one another, and on the graft upon those fragments.

Besides that of sepsis, usually introduced from without, though occasionally developed from a latent infection, hemorrhage is the chief risk which is associated with these operations. This can be best avoided by the use of very powerful hemostatic forceps, which are left in position in the wound for as long as possible during the course of the operation.

The relative value of security to convenience has not received the attention it merits, if one can judge by the routine methods which have been adopted in this war.

In [considering the measures that should be adopted in severe wounds about the knee-joint, when much bone has been carried away, the surgeon is apt to argue that if he cannot obtain bony ankylosis between the femur and the tibia he should amputate the limb, in the belief that an artificial limb will afford his patient the best mode of progression. A rigid limb gives the possessor security at the cost of convenience.

In dealing with these injuries the surgeon should, therefore, be influenced by the circumstances of the patient in deciding as to the relative advantages of security and convenience in each individual case.

In a large proportion of the community the individual is better off with a flail joint suitably supported by an apparatus than with the rigid limb or with an artificial limb.

Another injury, which is by no means uncommon, is that of a compound comminuted fracture of the lower end of the shaft of humerus not involving the elbow joint, from which fragments of bone of some size may have been removed.

As the movements of pronation and supination are performed perfectly in the normal elbow joint, and it is impossible to obtain that of flexion and extension in the elbow joint, it would seem advisable to develop a false joint between the humeral fragments which will permit of movement around a transverse axis. Such a joint improves in its functional capacity steadily, if suitable exercises are performed with the object of perfecting it.

